# Thiol-Functionalized Covalent Organic Framework for Efficient Metal Ion Removal in Water Treatment

**DOI:** 10.3390/nano15080582

**Published:** 2025-04-11

**Authors:** Cristina Arqueros, Lorena Welte, Carmen Montoro, Félix Zamora

**Affiliations:** 1 Kleinscale, Avenida Ciudad de Valencia S/N Parque Comercial Vera Plaza, Vera-Playa, 04621 Almería, Spain; crisarqueros@gmail.com (C.A.); lorenawelte@kleinscale.com (L.W.); 2 Departament of Inorganic Chemistry, Universidad Autónoma de Madrid, 28049 Madrid, Spain; 3 Institute for Advanced Research in Chemical Sciences (IAdChem), Universidad Autónoma de Madrid, 28049 Madrid, Spain; 4 Condensed Matter Physics Center (IFIMAC), Universidad Autónoma de Madrid, 28049 Madrid, Spain

**Keywords:** covalent organic framework, water treatment, adsorption, processability, mixed-matrix membranes, composite beads

## Abstract

Advanced water treatment technologies must offer selective, efficient, and cost-effective contaminant removal. In this study, TPB-DMTP-COF-SH, prepared from 1,3,5-tris(4-aminophenyl)benzene (TPB) and 2,5-dimethoxyterephaldehyde (DMTP), was synthesized via a two-step method and applied for the adsorption of aluminum (Al^3+^), iron (Fe^2+^), and manganese (Mn^2+^) ions from water. Adsorption performance was influenced by pH, initial concentration, and contact time, with optimal pH values of 3 for Al^3+^, 8 for Fe^2+^, and 10 for Mn^2+^. The adsorption data followed the Langmuir isotherm model, yielding maximum capacities of 3.27 mg g^−1^ (Al^3+^), 8.5 mg g^−1^ (Fe^2+^), and 0.67 mg g^−1^ (Mn^2+^). Kinetic studies indicated a pseudo-second-order mechanism, suggesting chemisorption as the dominant process. Equilibrium adsorption was reached at 15 min for Al^3+^ and Mn^2+^ and 20 min for Fe^2+^. As a proof of concept, we demonstrate that this thiol-functionalized COF not only effectively removes metals but also offers enhanced processability into composite beads and membranes, making it a strong candidate for real-world water treatment applications. These findings highlight TPB-DMTP-COF-SH as a promising and scalable solution for water purification.

## 1. Introduction

Improving industrial processes to become more environmentally friendly is a global priority nowadays. Some targets include reducing production times, decreasing energy consumption, and minimizing the use of large amounts of chemicals. These factors translate into more efficient processes and lower operational costs. Particularly in the water treatment context, developing new technologies based on novel materials capable of achieving these objectives is of great significance. In this regard, Covalent Organic Frameworks (COFs), which can be integrated into existing filtration systems, represent a promising avenue for enhancing water treatment technologies.

COFs are 2D crystalline materials composed of covalent bonds formed using light elements such as B, C, N, H, and O atoms, resulting in highly robust structures characterized by permanent porosity, high stability, and remarkable structural integrity [1,2]. Due to their derived properties, COFs can participate in a wide range of applications, including gas capture and separation [3], drug delivery [4], catalysis [5,6], and removal of contaminants from water [7,8], among others.

The affinity of a COF towards the capture of specific species and contaminants derives from the nature of the introduced functional group within the framework. Therefore, careful consideration and selection of the most suitable functionalization and route are crucial to avoid compromising the structural integrity of the precursor. In this sense, the preparation of brand-new frameworks endowed with different functionalities is being explored by post-synthetic modifications (PSMs) of already prepared materials [9]. There are multiple approaches to modifying formerly synthesized COFs, such as incorporating metals via coordination chemistry [10], monomer exchange [11,12], or chemical conversion of linkages by forming covalent bonds between a group already present in the framework with incoming constituents [13].

Many studies report post-synthetically modified COFs dotted with improved properties for water remediation purposes. For example, in 2019, Valtchev and co-workers reported the synthesis of a poly aryl ether-based COF, JUC-505, and its post-synthetic functionalization with carboxyl or amino functional groups yielding JUC-505-COOH and JUC-505-NH_2_. The COFs were obtained by refluxing the parent framework in sodium hydroxide (NaOH, 20% in ethanol/water, 1:1) and lithium aluminum hydride (LiAlH_4_), via nitrile hydrolysis and reduction, respectively. Both materials showed exceptional uptake of tetracycline antibiotics from water in a wide pH range (1–13) [14]. Later, Valtchev also reported introducing open carboxylic acid groups on a hydroxy-functionalized 3D COF via the ring-opening reaction with succinic anhydride. The carboxyl-functionalized material, 3D-COOH-COF, displayed high metal loading capacities and excellent adsorption selectivity for Nd^3+^ over Sr^2+^ and Fe^3+^ [15].

Very recently, the introduction of thiol groups in the pores has gained significance due to the exceptional binding ability of sulfur derivates with heavy metal ions (such as Hg^2+^ and Pb^2+^). However, these functional groups are not always compatible with COFs’ direct synthesis conditions, and a PSM route is more appropriate. This PSM is based on thiol-ene reactions where thiol groups are added to C=C under mild synthesis conditions. In this context, Ma et al. reported the post-synthetic modification of a new vinyl-functionalized COF, COF-V [16]. The vinyl pendant group allowed for the grafting of the thioether and thiols through a thiol-ene reaction with 1,2-ethanedithiol. The COF’s mercury removal was exceptional from both the aqueous media and gas phase, affording uptake capacities of 1350 and 863 mg g^−1^ for Hg^2+^ and Hg^0^, respectively. In the same year, Mancheño et al. reported a different thiol-grafted imine-based COF for Hg^2+^ removal present in water with the largest adsorption capacity to date, 4395 mg g^−1^ [17]. The material was first obtained through a copper-catalyzed azide–alkyne cycloaddition PSM and further incorporation of the thiol groups. The TPB-DMTP-COF-SH exhibited outstanding uptake capacity towards Hg^2+^, Sn^2+^, and Pb^2+^, but to a lesser extent. In addition, it was also found that TPB-DMTP-COF-SH showed high selectivity towards this ion even in the presence of high concentrations of other common metal ions such as Na^+^, Ca^2+^, Cu^2+^, Mg^2+^, and Zn^2+^.

Despite these remarkable results, the main limitation of COFs lies in that these materials are typically obtained in the form of insoluble powders, which significantly restricts their subsequent application under real operational conditions. Therefore, shaping COFs represents a significant challenge that must be addressed before their final application [18]. In this sense, Banerjee and collaborators were pioneers in preparing flexible, continuous, and crack-free self-standing COF membranes with thicknesses between 200 and 700 μm through the terracotta method [19]. COF dough containing reagents was knife-cast on a glass plate and further baked at 60–120 °C. This is an affordable and effective strategy to obtain COF membranes on a large scale with tunable thicknesses. Later, novel strategies for processing COFs have been developed, such as the preparation of aerogel monoliths [20,21] or membranes [22] for their implementation in water treatment purposes.

Taking this into account, we explore a novel application of the thiol-functionalized COF, TPB-DMTP-COF-SH, which has previously demonstrated remarkable uptake of heavy metal ions in water. In this work, we extend its use to the removal of ions that commonly cause incrustation and corrosion in water distribution systems, specifically Al^3+^, Mn^2+^, and Fe^2+^, an area that has received little attention. Additionally, we assess its processability through two strategies: encapsulating the powder within polysulfone to form composite beads and incorporating it as a filler in polymeric mixed matrix membranes (MMMs), paving the way for scalable integration into existing water treatment technologies.

## 2. Materials and Methods

### 2.1. Materials

All of the following chemicals and reagents were obtained from Carlo Erba Reagents S. A. (Sabadell, Barcelona, Spain) and utilized as received, without additional purification: copper iodide (CuI), dithiothreitol (DTT, Cleland’s reagent), *N*,*N*-diisopropylethylamine (DIPEA), tetrahydrofuran (THF), acetonitrile, triethylamine (TEA), dimethylformamide (DMF), isopropyl alcohol (IPA), aluminum chloride (AlCl_3_), iron(II) chloride tetrahydrate (FeCl_2_·4H_2_O), manganese(II) chloride (MnCl_2_), calcium chloride (CaCl_2_), magnesium dichloride (MgCl_2_), and arsenic trichloride (AsCl_3_).

The synthesis of [HC≡C]_0.5_-TPB-DMTP-COF [2] and 1,2-bis(2-azidoethyl)disulfane [1] was carried out following previously reported methodologies.

For the preparation of composite beads, polysulfone (PSU, Mw ~35,000) and N-methyl-2-pyrrolidone (NMP) were used, while polyvinylidene fluoride (PVDF, Mw ~275,000) was used for the fabrication of mixed-matrix membranes (MMMs). All reagents were sourced from Merk (Merck Life Science S.L.U., Madrid, Spain).

### 2.2. Synthesis of TPB-DMTP-COF-SH

The TPB-DMTP-COF-SH was synthesized by our previously described method, which is based on two steps [17]. In the first step, [HC≡C]_0.5_-TPB-DMTP-COF (200 mg, 0.16 mmol) and Cul (35 mg, 0.18 mmol) were added to a 100 mL two-neck round-bottom flask, and a solution of THF: H_2_O (11/4 mL) was added. The mixture was purged with Ar for 5 min, and DIPEA (200 µL) was added. Then, 1,2-bis(2-azidoethyl)disulfane (700 mg, 3.4 mmol) was added dropwise, and the suspension was stirred overnight at room temperature under an Ar atmosphere. The resulting solid, TPB-DMTP-COF-N_3_, was centrifugated at 4000 rpm for 4 min, washed with THF (3 × 40 mL) and acetonitrile (3 × 40 mL), and finally air dried.

In the second step, TPB-DMTP-COF-N_3_ (110 mg, 0.06 mmol) was suspended in a two-neck round-bottom flask in THF (2 mL). Under argon, DTT (53 mg, 0.34 mmol) and TEA (0.1 mL, 0.72 mmol) were added, and the mixture was stirred overnight at room temperature. The solid was collected by centrifugation at 4000 rpm for 4 min, washed with THF (3 × 40 mL) and acetonitrile (3 × 40 mL), and finally air dried. The material was activated at 120 °C under vacuum overnight, affording the product as a dark brown solid (η = 95%).

### 2.3. Preparation of TPB-DMTP-COF-SH@PSU Composite Beads

The composite TPB-DMTP-COF-SH@PSU beads were prepared as described below. In a 20 mL vial, 100 mg of TPB-DMTP-COF-SH powder and 4 g of a 10 wt/wt % PSU/NMP solution were mixed while stirring at 60 °C overnight. Beads were prepared with a 20 wt % loading of COF relative to the PSU polymer. The polymer–COF mixture was sonicated for complete homogenization and then added dropwise to a water bath at 0 °C to form the beads. The composite beads formed were washed with distilled water to remove the NMP (5 × 100 mL). The solvent was exchanged with IPA (5 × 100 mL). After the washes were performed, the beads were dried under Ar-flow at 75 °C. Finally, the beads were vacuum-activated at 100 °C overnight to remove any retained solvent.

### 2.4. Preparation of the TPB-DMTP-COF-SH@PVDF MMMs

In total, 5, 10 and 15 wt % PVDF stock solutions in DMF were prepared by dissolving the polymer in the organic solvent with sonication at 50 °C (see Table S14 for more details). TPB-DMTP-COF-SH@PVDF MMMs of 5, 10, and 15% wt/wt COF/PVDF loadings were obtained by adding the COF powder with the stock solution under stirring at 60 °C until complete homogenization of the mixture. The membrane-casting solution was poured into a Petri dish, followed by the evaporation of the solvent at 50 °C. The Petri dish was covered with a funnel to ensure slow removal of the solvent. After cooling to room temperature, the membrane was peeled off the glass and immersed in water.

### 2.5. Characterization of the COFs

Powder X-ray diffraction (PXRD) patterns were obtained using a Bruker D8 Advance X-ray diffractometer with Cu-Kα radiation (λ = 1.5418 Å) and a Lynxeye detector. Samples were prepared on a flat sample holder, and data were collected over a 2θ range of 2–40° with a step size of 0.03° and an exposure time of 1.3 s per step. Fourier-transform infrared spectroscopy (FTIR) measurements were acquired with a Perkin Elmer Spectrum 100 spectrometer equipped with a PIKE Technologies MIRacle single-reflection horizontal ATR accessory. The spectra were recorded over a range of 4000–400 cm^−1^. Solid-State ^13^C Cross-polarization with Magic Angle Spinning (^13^CP-MAS) NMR spectra were obtained at room temperature on a Bruker AV 400 WB spectrometer with a triple-channel, 4 mm probe and zirconia rotors sealed with Kel-F caps. Spectra were acquired at 100.61 MHz using ^13^CP-MAS, with chemical shifts reported in parts per million (ppm, δ scale) relative to tetramethylsilane (TMS) as the reference. Elemental analysis (EA) was determined with a LECO CHNS-932 analyzer. Thermogravimetric analyses (TGAs) were performed using a TA Instruments Q500 thermogravimetric analyzer. Samples were placed in an aluminum pan and analyzed under a N_2_ atmosphere. The temperature was ramped from 25 °C to 1000 °C at 10 °C per minute. Scanning Electron Microscopy (SEM) imaging was conducted on a JEOL JSM 6335F microscope at an accelerating voltage of 15 kV. Samples were dispersed on a conductive adhesive mounted on a copper platform and coated with a 12 nm chromium layer using a Quorum Q150T-S sputter coater. N_2_ adsorption–desorption isotherms at 77 K were measured on a Micromeritics ASAP2020 volumetric analyzer under static conditions. Samples were pre-treated by heating to 120 °C overnight and outgassed to 10^−6^ Torr. The specific surface area (S_BET_) and pore volume were calculated using the Brunauer–Emmett–Teller (BET) and Langmuir models. In contrast, the pore size distribution (PSD) was determined using the non-local density functional theory (NLDFT) method based on the model N_2_ at 77 K on carbon cylinder pores via Microactive software v4.04. The ion content was analyzed using the HI-83300 photometer from the Hanna instrument or by inductively coupled plasma mass spectrometry (ICP-MS) using a ThermoScientific iCAP TQ ICP-MS system.

### 2.6. Adsorption Experiments

The adsorption experiments involved exposing the TPB-DMTP-COF-SH to an aqueous solution with a known concentration of the target ion to be removed (see Table S2). After a specific contact time, the material was removed, and the final concentration was measured by colorimetric photometry or with an ICP-MS to gain more precision. As a first approach, the adsorption capacity of the COF toward the metal ions Al^3+^, Ca^2+^, Mg^2+^, Mn^2+^, Fe^2+^, and As^3+^ was studied. The removal efficiency and adsorption capacity (Q_e_, mg L^−1^) in the batch experiment were calculated with the following equations:

(1) $$Removal\;efficiency=\frac{\left(C_{i}-C_{f}\right)}{C_{i}}\times 100\%$$

(2) $$Q_{e}=\frac{{(C}_{i}-C_{f})\cdot V}{m}$$

where *C_f_* (mg L^−1^) is the final concentration of the liquid phase, *C_i_* (mg^−1^ L) is the initial concentration, *V* (L) is the sample volume, and *m* (g) is the mass of the adsorbent.

The removal efficiency of the beads was evaluated for the removal of Al^3+^, Fe^2+^, and Mn^2+^, with the solution containing 0.3, 0.65, and 0.25 mg L^−1^ concentrations, respectively. Around 65 mg of beads were weighed and put in contact with each solution for 1 h; then, the beads were separated, and the final concentration was measured via ICP-MS.

#### 2.6.1. Effect of pH

The pH was adjusted to 3, 5, 7, and 10 with 0.1 M NaOH and 0.1 M HCl solutions, and in the case of iron was also measured at pH 12. The COF powder was put in contact with the sample for 1 h with stirring at 200 rpm and then filtered through a 0.45 µm PVDF syringe filter to remove the powder and measure the final concentration.

#### 2.6.2. Adsorption Isotherms

The maximum adsorption capacity of TPB-DMTP-COF-SH was assessed by putting around 10 mg of the material in contact with aqueous solutions of Al^3+^ (0–1.5 mg L^−1^, pH 6), Fe^2+^ (0–4 mg L^−1^, pH 8), and Mn^2+^ (0–1.7 mg L^−1^, pH 8). The samples were shaken at 200 rpm for 1 h at 25 °C. After that, the solution was filtered through a 0.45 μm PVDF syringe filter to remove the COF, and the final concentration was analyzed via ICP-MS. There are numerous adsorption models, such as the Langmuir, Freundlich, Dubinin–Radushkevich, Henry, Temkin models, etc., that have been developed to analyze experimental data and predict the nature of adsorption (monolayer, multilayers, or homogeneous/heterogeneous). Langmuir and Freundlich’s adsorption isotherm models are the most commonly used for removing contaminants from wastewater.

The Langmuir isotherm model is expressed with the following mathematical equations:

Non-linear equation:

(3) $$Q_{e}=Q_{max}\cdot \frac{C_{e}\cdot K}{\left(1+K_{L}\cdot C_{e}\right)}$$

where *Q_e_* (mg g^−1^) is the quantity adsorbed in the equilibrium, *C_e_* (mg L^−1^) is the equilibrium concentration of the liquid phase, *K_L_* (L mg^−1^) is the Langmuir affinity constant, and *Q_max_* (mg g^−1^) is the maximum capacity of the adsorbent.

To calculate the Langmuir isotherm parameters, Equation (3) can be expressed into two different linear-form equations. The first linear Langmuir isotherm equation can be described as Equation (4) by plotting *C_e_*/*Q_e_* against *C_e_*.

Linear Langmuir-1:

(4) $$\frac{C_{e}}{Q_{e}}=\frac{1}{K_{L}\cdot Q_{max}}+\frac{1}{Q_{max}}\cdot C_{e}$$

The second linear Langmuir isotherm can be written as Equation (5), by plotting 1/*Q_e_* versus 1/*C_e_*.

Linear Langmuir-2:

(5) $$\frac{1}{Q_{e}}=\frac{1}{Q_{max}}+\frac{1}{\left(K_{L}\cdot Q_{max}\right)}\cdot \frac{1}{C_{e}}$$

The Freundlich isotherm model is expressed with the following mathematical equations:

Non-linear equation:

(6) Qe=KF·Ce1n

where *Q_e_* (mg g^−1^) is the quantity adsorbed in the equilibrium, *C_e_* (mg L^−1^) is the equilibrium concentration of the liquid phase, 1/*n* gives the intensity of the adsorbate adsorption, and *K_F_* (L g^−1^) is the Freundlich affinity constant.

The linear Freundlich isotherm can be written as Equation (7), by plotting *ln*(*Q_e_*) versus *ln*(*C_e_*).

(7) $$ln\left(Q_{e}\right)=\frac{1}{n}\cdot \ln\left(C_{e}\right)+ln(K_{F})$$

The Temkin isotherm model is expressed with the following mathematical equations:

Non-linear equation:

(8) $$Q_{e}=\frac{R\cdot T}{b_{T}}\cdot \ln\left(K_{T}\cdot C_{e}\right)$$

where *Q_e_* (mg g^−1^) is the quantity adsorbed in the equilibrium, *T* is the temperature at 298 K, *R* is the universal gas constant (8.314 J mol^−1^ K^−1^), *b_T_* and *K_T_* are the Temkin isotherm constant and equilibrium binding constant (g^−1^), respectively, and, *C_e_* (mg L^−1^) is the equilibrium concentration of the liquid phase.

The linear Temkin isotherm can be written as Equation (7), by plotting *Q_e_* versus *ln*(*C_e_*).

(9) $$Q_{e}=\frac{R\cdot T}{b_{T}}\cdot \ln\left(K_{T}\right)+\frac{R\cdot T}{b_{T}}\cdot ln(C_{e})$$

#### 2.6.3. Adsorption Kinetics

A total of 10 mg of TPB-DMTP-COF-SH powder was mixed with 10 mL of a solution containing a fixed initial concentration of each ion, 1.5 ppm Al^3+^, 1 ppm Fe^2+^, and 0.5 ppm Mn^2+^. The samples were shaken at 200 rpm with different contact times (t = 1, 3, 5, 10, 15, 30, 60, 120, and 250 min). For each time interval, the same amount of COF was added to a solution with a fixed initial ion concentration, and all mixtures were shaken simultaneously for different contact times. The samples were then filtered through a 0.45 µm PVDF syringe filter to remove the COF, and the final concentration was analyzed via ICP-MS.

The adsorption rate of metal ions at low concentrations in water was studied using pseudo-first-order and pseudo-second-order kinetics [23].

The pseudo-first-order kinetic model is described with the following equations [24]:

Non-linear equation:

(10) $$Q_{t}=Q_{e}\cdot (1-e^{-kt})$$

Linear form:

(11) $$\log\left(Q_{e}-Q_{t}\right)=\log Q_{e}-(\frac{k_{1}}{3.03})\cdot t$$

where *Q_t_* (mg g^−1^) is the quantity adsorbed at a determined t (min), and *Q_e_* (mg g^−1^) is the quantity adsorbed at equilibrium. *k*_1_ (g min^−1^ mg^−1^) is the pseudo-first-order rate constant.

The pseudo-second-order kinetic model is described with the following equations [24]:

Non-linear equation:

(12) $$Q_{t}=\frac{k_{2}{\cdot Q}_{e}^{2}\cdot t}{\left(1+k_{2}\cdot Q_{e}\cdot t\right)}$$

Linear form:

(13) $$\frac{t}{Q_{t}}=\frac{1}{k_{2}{\cdot Q}_{e}^{2}}+\frac{1}{Q_{e}}\cdot t$$

where *Q_t_* (mg g^−1^) is the quantity adsorbed at a determined *t* (min), and *Q_e_* (mg g^−1^) is the quantity adsorbed at equilibrium. *k*_2_ (g min^−1^ mg^−1^) is the pseudo-second-order rate constant.

#### 2.6.4. Permeation

The permeation of the membranes was first tested by measuring the volumetric flux, *J* (L m^−2^ h^−1^ or LMH). It is the solvent volume (in this case, distilled water) collected from the permeate per unit of membrane area per unit of time [25].

(14) $$J=\frac{V}{A\cdot t}$$

where *V* (L) is the volume, *A* (m^2^) is the area, and *t* (h) is the time. The *J* of all the prepared membranes was measured using a Sterlitech HP4750 High-Pressure Stirred Cell (see Scheme S6.1) [26]. The performance of the membranes in terms of flux was determined using an area reducer of Ø = 1.32 cm. Most of the membranes were subjected to a constant pressure of 2 bar, while two underwent a pressure of 1 bar. The experiments were carried out at room temperature.

## 3. Results and Discussion

### 3.1. Synthesis and Characterization of TPB-DMTP-COF-SH

**Scheme 1:**
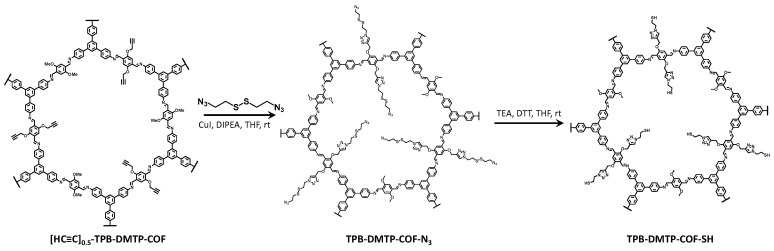
Procedure for the synthesis of TPB-DMTP-COF-SH.

TPB-DMTP-COF-SH was synthesized by a two-step reaction method following the procedure we previously reported (Scheme 1) [17]. First, the precursor [HC≡C]_0.5_-TPB-DMTP-COF was post-synthetically functionalized to incorporate triazole groups in its pore via “click chemistry” with 1,2-bis (2-azidoethyl)disulfane in basic media, THF, and DIPEA. This reaction is based on the copper (I)-catalyzed azide–alkyne cycloaddition or Huisgen’s cycloaddition [27]. Then, the incorporation of thiol groups was carried out to the product obtained, TPB-DMTP-COF-N_3_, using the Clealand reagent (DTT), also in basic media, THF and TEA, to yield the final COF material, TPB-DMTP-COF-SH, which was activated at 120 °C under vacuum.

**Figure 1:**
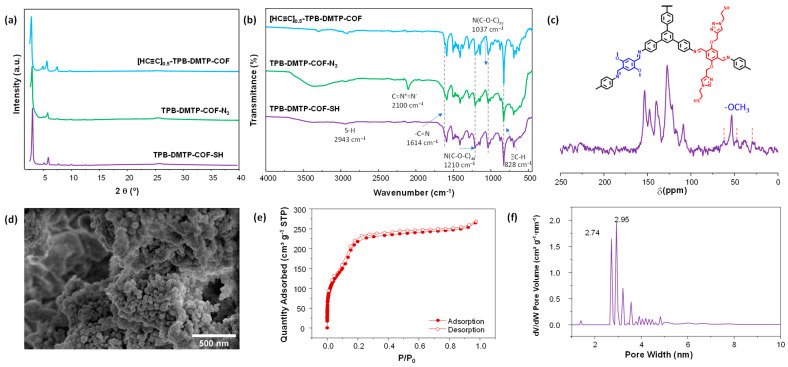
(**a**) PXRD patterns and (**b**) FTIR spectra of the precursor [HC≡C]_0.5_-TPB-DMTP-COF, the intermediate TPB-DMTP-COF-N_3_, and the final product TPB-DMTP-COF-SH. (**c**) Solid-state ^13^C CP-MAS NMR spectra, (**d**) SEM image, (**e**) N_2_ adsorption–desorption isotherms, and (**f**) pore size distribution of TPB-DMTP-COF-SH.

To confirm the successful preparation of the TPB-DMTP-COF-SH compared to its precursors, it was characterized in terms of its structural, morphological, and textural properties using various characterization techniques. Firstly, the crystallinity was confirmed through powder X-ray diffraction (PXRD). The obtained diffraction patterns verified that the COF’s crystallinity was successfully preserved after the two-step post-synthetic functionalization process (Figure 1a) [17]. The formation of the imine bond and the incorporation of the functional groups in the pores of the framework were corroborated via Fourier-transform infrared spectroscopy (FTIR) and solid-state ^13^C cross-polarization with magic-angle spinning (^13^CP-MAS) NMR spectroscopy. The presence of the *υ*(C=N) signal at 1614 cm^−1^ indicates the correct formation of the imine-based framework (Figure 1b). In the comparative spectra, the apparition of the azide peak at 2100 cm^−1^ in the intermediate material and its disappearance in the final product can be noticed, confirming the incorporation of the thiol groups, as well as the signal at 2943 cm^−1^ that corresponds to the *υ*(S-H) stretching. The solid-state ^13^C CP-MAS NMR spectra analyzed the chemical structures of the product (Figure 1c). The signals of the C sp^2^ appear between 100 and 150 ppm, corresponding to the imine bond. The signal at 53.2 ppm belongs to the methoxy groups, and the signals at 62.0, 47.1, and 29.6 ppm correspond to the aliphatic chains connecting the thiol groups to the triazoles in the COF. The results from elemental analysis (see Table S1) and thermogravimetric analysis (TGA) (see Figure S5) are provided and discussed below. Particularly, TGA analysis shows that the material is stable up to 800 °C, with a total mass loss of 41%, primarily due to the evaporation of solvent traces used during synthesis (such as H_2_O, THF, Toluene, and DIPEA). Regarding the CHN analysis, the main objective was to confirm the functionalization of the material with the thiol group. The results show a significant increase in sulfur content, from 0.1% in [C≡C]_0.5_-TPB-DMTP-COF to 7.2% in TPB-DMTP-COF-N_3_ and 6.2% in TPB-DMTP-COF-SH, confirming the successful incorporation of the thiol group into the material’s structure. These results demonstrate that the composition of the synthesized material and its thermal stability are consistent with the expected values, further validating the success of the synthesis process and the material’s suitability for the intended applications. The morphology of the synthesized COF was studied through scanning electron microscopy (SEM). SEM images for TPB-DMTP-COF-SH revealed small particles with sizes around 30–40 nm and formed larger aggregates (Figure 1d).

N_2_ adsorption–desorption isotherms assessed the textural properties of TPB-DMTP-COF-SH at 77 K. The isotherm exhibited a Type IV shape (Figure 1e), characteristic of mesoporous materials, and the S_BET_ obtained was 1090 m^2^ g^−1^ [17]. PSD revealed two main pore sizes of 2.74 and 2.95 nm (Figure 1f). The presence of some pores with larger sizes could be attributed to the formation of defects.

### 3.2. Adsorption Properties of TPB-DMTP-COF-SH

**Figure 2:**
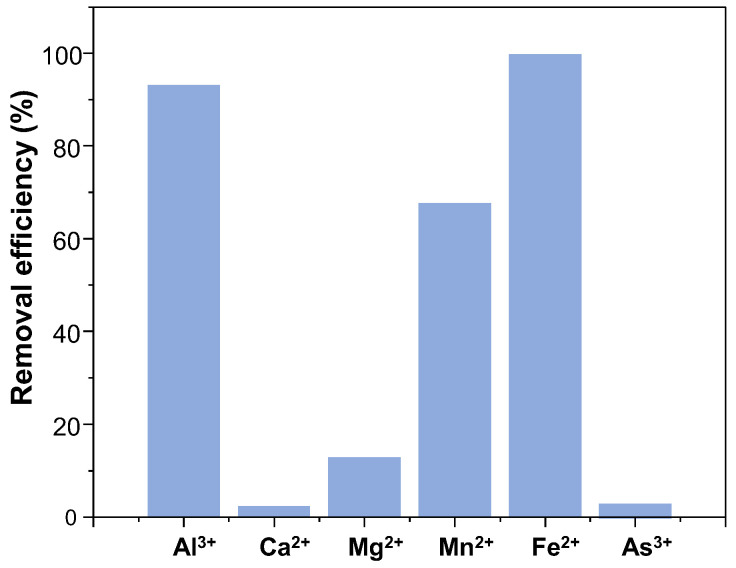
Removal efficiency was obtained in the general scope in adsorption studies of TPB-DMTP-COF-SH for the proposed ions. Experimental results were measured at 25 °C, 15 min, pH = 6, and the initial concentrations are C_i,Al3+_ = 0.36 mg^−1^ L, C_i,Ca2+_ = 34.20 mg^−1^ L, C_i,Mg2+_ = 19.81 mg^−1^ L, C_i,Mn2+_ = 0.30 mg^−1^ L, C_i,Fe2+_ = 0.22 mg^−1^ L, C_i,As3+_ = 1.02 mg^−1^ L.

The adsorption properties of the prepared COF, TPB-DMTP-COF-SH, were studied to capture metal ions from water. Notably, we have examined the material’s performance in the adsorption of Al^3+^, Ca^2+^, Mg^2+^, Mn^2+^, Fe^2+^, and As^3+^, which are toxic and contribute to fouling and corrosion events in industrial plants. This study was carried out by mixing a controlled amount of COF powder with solutions containing a determined ion concentration (see Table S2). The concentration range was chosen based on typical metal ion levels in water, considering EU drinking water limits and the material’s saturation capacity. After a certain period, the material was removed, and the final metal ion concentration was measured by colorimetric photometry, based on single measurements. The COF showed huge removal efficiencies of 99% and 93% for iron and aluminum, respectively (Figure 2). It also achieved good retention of manganese, with 68% removal. Due to this exceptional removal efficiency, we have carried out a deeper study of the adsorption properties of the material towards Fe^2+^, Al^3+^, and Mn^2+^.

#### 3.2.1. Effect of pH

**Figure 3:**
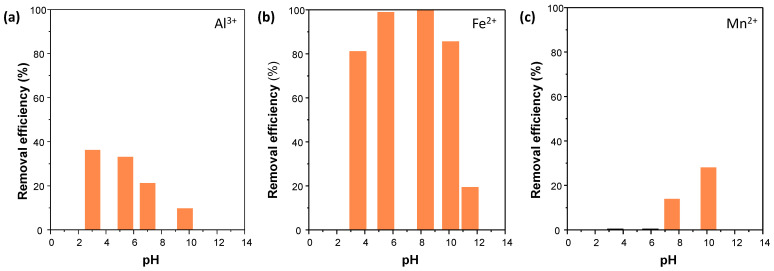
Removal efficiencies of TPB-DMTP-COF-SH towards (**a**) Al^3+^, (**b**) Fe^2+^, and (**c**) Mn^2+^ at different pH values.

The adsorption capacity for each metal ion was studied as a function of the media’s pH. Four samples with different pH values (3, 5, 7, and 10) were prepared for aluminum, iron, and manganese studies, and the data were obtained from single measurements. The uptake of Al^3+^ in the COF adsorbent increased at lower pH values, as it is the main aluminum species and most soluble in acidic media. Under neutral conditions, insoluble aluminum hydroxide (Al(OH)_3_) is dominant, whereas, in alkaline conditions, it exists predominantly in the form of Al(OH)_4_^−^ (Figure 3a and Table S3). In the case of iron, the uptake revealed significant retention rates at a wide range of pH values, with a severe decrease at pH 11.5 (Figure 3b and Table S4). Under oxidizing and alkaline conditions, iron tends to oxidize from Fe^2+^ to Fe^3+^, forming insoluble complexes of iron hydroxides, resulting in a direct decrease in the adsorption capacity of the COF towards this ion [28,29]. Finally, the adsorption of Mn^2+^ increased at higher pH values (Figure 3c and Table S5), agreeing with the initial theory, in which a negatively charged surface would enhance electrostatic interactions between the thiol groups of the pores and the manganese cation [17,18].

#### 3.2.2. Adsorption Isotherms

**Figure 4:**
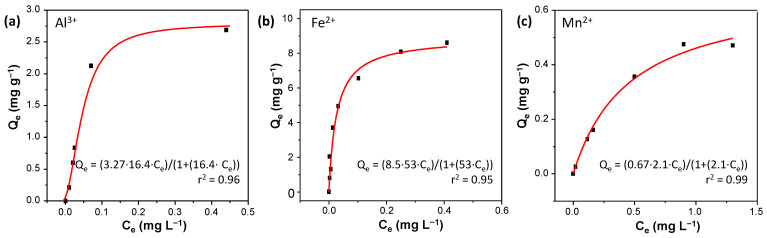
Adsorption isotherms of TPB-DMTP-COF-SH towards (**a**) Al^3+^, (**b**) Fe^2+^, and (**c**) Mn^2+^.

The studies were conducted under constant temperature and pH conditions, with the initial concentration as the only variable parameter. To identify the most suitable adsorption isotherm model for describing and optimizing the adsorption process, the experimental data were analyzed using the Langmuir, Freundlich, and Temkin models in both their linear and non-linear forms (Figure 4 and Figures S6–S10). The isotherm parameters were estimated using least-squares regression to determine the best-fit equilibrium isotherm. Although linear regression is widely used due to its simplicity and the straightforward determination of fitting parameters from the slope and intercept, it has significant limitations. Linearization can distort the statistical interpretation of experimental errors, violate the assumption of constant error variance, and introduce inaccuracies in the independent variable. In contrast, non-linear regression, by iteratively adjusting fitting parameters to minimize the sum of squared errors, offers a more reliable and robust approach for adsorption modeling.

The Langmuir isotherm model assumes monolayer adsorption and enables the calculation of the saturation concentration of the sorbate in the adsorbent. While its nonlinear form can be transformed into various linear expressions, in this study, we employed the Linear Langmuir-1 equation due to its reliability and close agreement with the nonlinear fitting. The Freundlich isotherm model, on the other hand, describes multilayer and non-ideal adsorption on heterogeneous surfaces, while the Temkin isotherm model evaluates adsorbent–adsorbate interactions, assuming that the heat of adsorption decreases linearly with surface coverage, leading to a non-uniform distribution of adsorption heat and binding energies.

After analyzing the data, we concluded that the experimental results fit the Langmuir model better than the Freundlich or Temkin models, as indicated by the higher r² values (Table S9). Additionally, the Temkin isotherm provided a slightly better fit than the Freundlich isotherm. Furthermore, the correlation coefficients obtained from the nonlinear model were slightly higher than those from the linear model, reinforcing the advantages of using nonlinear regression for a more accurate representation of adsorption data.

The adsorption results obtained fit the Langmuir model [30] (Figure 4 and Tables S6–S8), indicating a maximum adsorption capacity *Q_max,Al_* = 3.27 mg g^−1^, *Q_max,Fe_* = 8.5 mg g^−1^, and *Q_max,Mn_* = 0.67 mg g^−1^. The Langmuir affinity (K_L_) constants were determined as *K_Al_* = 16 L mg^−1^, *K_Fe_* = 53 L mg^−1^, and *K_Mn_* = 2.1 L mg^−1^. These values highlight the strong affinity towards Al^3+^, Fe^2+^, and Mn^2+^ and reflect the extent of interaction between the adsorbate and the adsorption sites.

#### 3.2.3. Adsorption Kinetics

**Figure 5:**
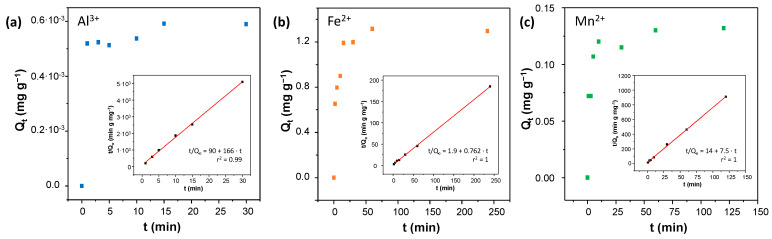
Adsorption kinetics of TPB-DMTP-COF-SH towards (**a**) Al^3+^, (**b**) Fe^2+^, and (**c**) Mn^2+^. Insets: fitting a pseudo-second-order linear model.

After determining the saturation concentration of each ion, the adsorption kinetics were analyzed to evaluate the adsorption rate, identify the controlling mechanisms of the process (e.g., diffusion or surface interactions), and assess the efficiency and performance of the adsorbent over time. For that, COF material from the same batch synthesis was used in separate vials. Identical amounts of COF were added to solutions with a fixed initial ion concentration, and all mixtures were shaken simultaneously for different contact times. This approach ensured study homogeneity and measurement accuracy while preventing material loss and experimental interference, thus improving data reliability. The data were fitted to pseudo-first-order (Figures S11–S13) and pseudo-second-order (Figure 5) kinetic models, with the latter showing better correlation coefficients (*r*^2^*_Al_* = 0.99868, *r*^2^*_Fe_* = 0.99970, and *r*^2^*_Mn_* = 0.99901). This result indicates that the adsorption process is predominantly governed by chemisorption involving electron sharing or exchange between the adsorbate and the adsorbent surface. The adsorption capacity (*Q_t_*) reached its maximum at 15, 20, and 15 min for Al^3+^, Fe^2+^, and Mn^2+^, respectively (Tables S10–S12). Lastly, the pseudo-second-order rate constant was obtained from the intercept of the linear equation *k*_2 *Al*_ = 308.14 g min^−1^ mg^−1^, *k*_2 *Fe*_ = 0.31 g min^−1^ mg^−1^, and *k*_2 *Mn*_ = 3.78 g min^−1^ mg^−1^.

After the retention studies with Al^3+^, Fe^2+^, and Mn^2+^, the material was recovered and subsequently analyzed via infrared spectroscopy to confirm its structural stability (see Figure S1). FTIR spectra of TPB-DMTP-COF-SH@Al, TPB-DMTP-COF-SH@Fe, and TPB-DMTP-COF-SH@Mn demonstrated that the structure of the framework was retained, and no new bonds were formed.

### 3.3. Processing COFs

**Figure 6:**
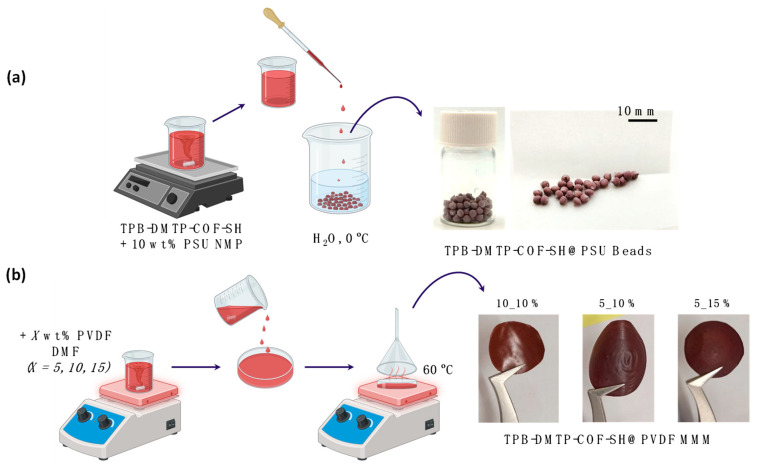
(**a**) Scheme of the procedure preparation of TPB-DMTP-COF-SH@PSU composite beads and (**b**) COF-based MMMs.

One of the main limitations of COFs when considering their implementation in real applications is that most of them are prepared as polycrystalline powder. In this work, given the results obtained for the TPB-DMTP-COF-SH in the capture of Al^3+^, Fe^2+^, and Mn^2+^ from water, a preliminary study of the material’s processability in the form of composite beads and hybrid membranes or mixed-matrix membranes (MMMs) has been conducted (Figure 6).

#### 3.3.1. Preparation and Performance of Composite Beads

Composite beads were fabricated by introducing a PSU solution containing 20 wt % COF into cold water through a droplet formation process. FTIR analysis confirmed the incorporation of COF into the composite beads (Figure S2). The formed beads displayed diameters ranging from 2 to 3 mm and possessed onion-like shapes (Figure 6).

The removal efficiency of the beads was evaluated for the removal of Al^3+^, Fe^2+^, and Mn^2+^ from water. The beads removed up to 47% of the Al^3+^ concentration in water and 31 and 24% for Fe^2+^ and Mn^2+^, respectively (see Table S13). The adsorption capacity of the beads was calculated by taking into account only the COF mass present in the composite beads (20 wt %). The adsorption capacities obtained, *Qe*, were slightly lower than those in the powder retention studies. This may be due to poor cation diffusion through the polymer coating and the limited surface area within the composite beads. Further optimization in preparing smaller beads could be worthwhile in achieving enhanced surface areas and excellent retention rates.

#### 3.3.2. Preparation and Performance of MMMs

**Figure 7:**
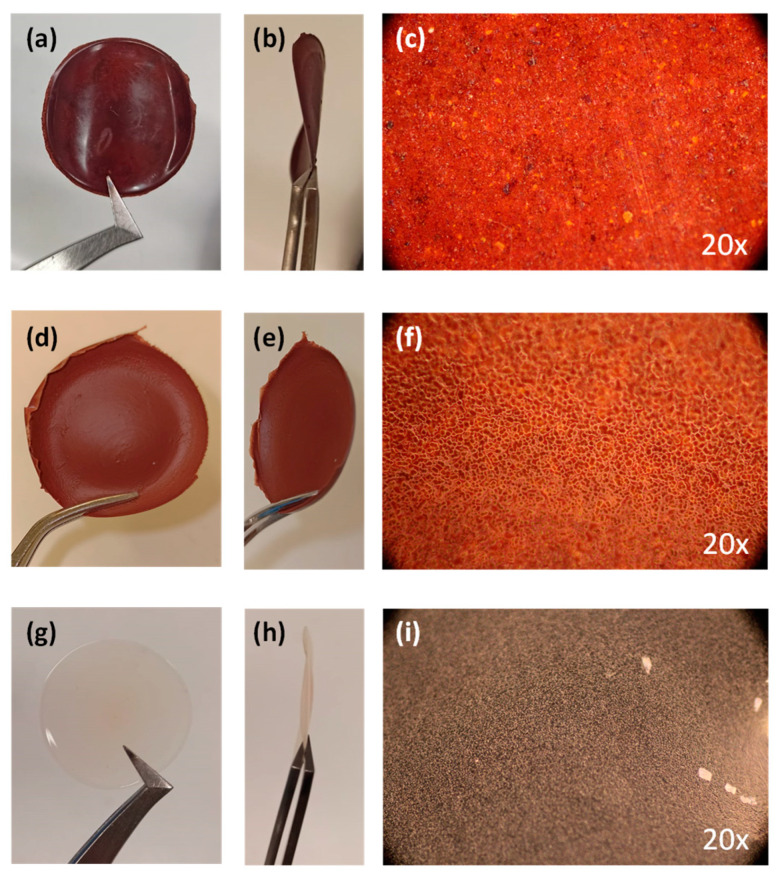
Photographs of the COF@PVDF MMMs: TPB-DMTP-COF-SH@PVDF 5_5% wt/wt (**a**) front view and (**b**) side view; TPB-DMTP-COF-SH@PVDF 10_5% wt/wt (**d**) front view and (**e**) side view; control PVDF (**g**) front view and (**h**) side view. Optical images of (**c**,**f**) the COF membranes and (**i**) the PVDF polymer.

TPB-DMTP-COF-SH was also processed as hybrid membranes or mixed-matrix membranes (MMMs). MMMs were prepared with different wt % COF loading by mixing the COF powder with a stock solution of PVDF in DMF prepared beforehand. Membranes were denoted as *wt % filler*_*wt % polymer*. The MMM with 5 wt % loading of COF in 5 wt % PVDF, DMTP-COF-SH@PVDF 5_5 wt/wt % exhibited very low thickness (0.13 mm) and showed high permeation without any significant applied pressure (Figure 7a–c). The MMM with 10 wt % PVDF, TPB-DMTP-COF-SH, showed greater consistency (0.24 mm thickness) and good flexibility (Figure 7d–f). Moreover, a control polymer membrane consisting solely of PVDF was prepared, yielding a membrane with 0.18 mm thickness (Figure 7g–i).

Color comparison between the control membrane and the MMMs showed the incorporation of TPB-DMTP-COF-SH into the polymeric matrix. TPB-DMTP-COF-SH@PVDF 5_5 and 10_5% wt/wt MMMs display good homogeneity through optical microscopy (Figure 7c,f). However, membranes loaded with 10 wt % COF exhibited reduced flexibility and were more brittle than those with 5% loading. An optimal COF/polymer ratio of less than 1 was established. Polymer loading was increased to 10% and 15% to achieve higher COF loadings, resulting in membranes with 5_10%, 10_10%, and 5_15% loadings (Table S14).

The presence of COF in the polymer matrix was confirmed by FTIR through structural characterization of the membranes. The spectra of the membranes exhibited slight observation of the characteristic peaks of the COF corresponding to the S-H and -C=N stretching at 2943 and 1614 cm^−1^, respectively (Figure S3). Furthermore, these peaks become more significant as the COF/PVDF ratio increases (Figure S4). The polymeric matrix signals are observable in all the prepared membranes, with particular attention given to the 1231 cm^−1^ band, characteristic of the gamma (γ) polymer phase [31].

As a proof of concept of the membrane performance, the permeation of the membranes was tested by measuring the volumetric flux, J (L m^−2^ h^−1^ or LMH). J is the solvent volume (in this case, distilled water) collected from the permeate per unit of membrane area per unit of time [25]. Achieving a balance between rejection and volumetric flux is critical in membrane technology to prevent excessive operating pressures and costs. Several factors impact the operating pressures, including the membrane pattern, thickness, filler material, choice of polymer matrix, and loading.

PVDF control membranes displayed fluxes of 438, 350, and 175 LMH, which decreased significantly with increasing % PVDF loading from 5 to 10 and 15% wt, respectively (Table S15). Adding COF filler to the 5 wt % PVDF membrane resulted in a substantial increase in water flux from 438 to 1926 and 3289 LMH for 5 and 10% COF loadings, respectively. Furthermore, incorporating COF filler into the 10 wt % PVDF membranes increased flux from 350 to 613 and 525 LMH for 5 and 10% wt/wt COF loadings, respectively. In addition, the increase in PVDF loading resulted in a direct decrease in water flux of 68% when duplicating the loading from 5 to 10 wt % of PVDF in 5% wt/wt COF membranes. Similarly, MMMs with 10% wt/wt COF showed a decrease of 84% when duplicating the % PVDF loading. In conclusion, as expected, increasing the loading of PVDF leads to a reduction in the permeation flux of the membranes, and higher loading of fillers leads to an increase in the flux.

The water permeability values of the prepared MMMs agree with previously reported studies using COFs as fillers [32]. However, these values are still quite large considering average operation fluxes range from 10 to 200 L m^−2^ h^−1^ in water filtration processes using membranes with other porous fillers such as TiO_2_ NPs [33], MOFs [34,35], multi-walled carbon nanotubes (MWCNTs) [36,37], zeolite imidazolate frameworks (ZIFs) [38], silica [39], or carbon molecular sieves [40] (Figure S14). The flux values of the MMMs COF-based membranes need to be optimized to reach around 200 L m^−2^ h^−1^ in water, after which adsorption tests towards cations will be performed to assess the technology’s effective applicability.

## 4. Conclusions

A covalent organic framework (COF) material was synthesized via post-synthetic modification using Huisgen’s cycloaddition (“click chemistry”), followed by thiol functionalization to obtain TPB-DMTP-COF-SH. The material was structurally, texturally, and morphologically characterized and evaluated for the removal of common corrosive and fouling metal ions (Ca^2+^, Mg^2+^, Mn^2+^, Fe^2+^, Al^3+^, and As^3+^). Notably, it exhibited strong affinity for Mn^2+^, Fe^2+^, and Al^3+^, which were further investigated. The COF demonstrated maximum adsorption capacities of 3.27 mg g^−1^ for Al^3+^, 8.5 mg g^−1^ for Fe^2+^, and 0.67 mg g^−1^ for Mn^2+^, with adsorption kinetics following a pseudo-second-order model and rapid retention rates.

The material was successfully processed into COF@PSU composite beads and COF@PVDF mixed-matrix membranes (MMMs). The adsorption performance of COF@PSU beads was evaluated for cation removal, while MMMs with varying COF loadings were structurally characterized and tested for flux measurements. This study highlights the potential of COFs for water treatment applications, offering an alternative to conventional technologies such as coagulation/flocculation, sedimentation, and oxidation. More importantly, our work advances the field by demonstrating a successful strategy to process COFs into practical, scalable forms suitable for real-world applications. Additionally, COF-based materials could be an effective pretreatment step before advanced membrane filtration processes like nanofiltration or reverse osmosis.

## Data Availability

The data presented in this study are available in the article and in the Supplementary Materials.

## Supplementary Materials

The following supporting information can be downloaded at https://www.mdpi.com/article/10.3390/nano15080582/s1. Figures S1–S4. FTIR spectra of TPB-DMTP-COF-SH@Al, TPB-DMTP-COF-SH@Fe, TPB-DMTP-COF-SH@Mn, TPB-DMTP-COF-SH, Polysulfone, TPB-DMTP-COF-SH@PSU composite beads and the prepared MMMs; Table S1. Composition of TPB-DMTP-COF-SH obtained by EA; Figure S5. TGA profile of TPB-DMTP-COF-SH; Table S2. Experimental data of the adsorptive properties of TPB-DMTP-COF-SH towards different contaminants; Tables S3–S5. Data from the pH dependence study in the removal of Al^3+^, Fe^2+^, and Mn^2+^ ions of TPB-DMTP-COF-SH; Tables S6–S9 and Figures S6–S10. Data from the adsorption isotherm of TPB-DMTP-COF-SH towards Al^3+^, Fe^2+^, and Mn^2+^. Tables S10–S12 and Figures S11–S13. Experimental results from the adsorption kinetic of TPB-DMTP-COF-SH towards Al^3+^, Fe^2+^, and Mn^2+^; Table S13. TPB-DMTP-COF-SH@PSU beads’ adsorption performance towards the selected ions; Table S14. PVDF and COF quantities for the preparation of the different MMMs; Scheme S1. Scheme of the Sterlitech HP4750 high-pressure stirred cell; Table S15. Volumetric fluxes of the prepared membranes and characteristics; Figure S14. Comparison of operating flows with other porous materials.
