# Supplementary material for: Thiol-Functionalized Covalent Organic Framework for Efficient Metal Ion Removal in Water Treatment
Source: Nanomaterials (Basel). 2025 Apr 11;15(8):582. doi: 10.3390/nano15080582 (PMC12029346; doi:10.3390/nano15080582)
Supplement: Supplementary file 1 [file nanomaterials-15-00582-s001.zip › nanomaterials-3521875-supplementary.pdf]

Supplementary Information

Thiol-Functionalized Covalent Organic Framework for Efficient Metal Ion Removal in Water Treatment

Cristina Arqueros [1], Lorena Welte [1], Carmen Montoro [2,3,*] and Félix Zamora [2,4,*]

[1.] Kleinscale, Avenida Ciudad de Valencia S/N Parque Comercial Vera Plaza, Vera-Playa, 04621 Almería, Spain
[2.] Departament of Inorganic Chemistry, Universidad Autónoma de Madrid, 28049 Madrid, Spain.
[3.] Institute for Advanced Research in Chemical Sciences (IAdChem), Universidad Autónoma de Madrid, 28049 Madrid, Spain; carmen.montoro@uam.es
[4.] Condensed Matter Physics Center (IFIMAC), Universidad Autónoma de Madrid, 28049 Madrid, Spain; felix.zamora@uam.es

Table of Contents

1. Fourier-Transform Infrared Spectroscopy (FTIR) 3

2. Elemental Analysis (EA) 5

3. Thermogravimetric Analysis (TGA) 5

4. Additional data on the adsorption performance 6

5. Preparation and performance of composite beads 13

6. Preparation and performance of TPB-DMTP-COF-SH@PPVDF MMMs 13

7. References 15

1. Fourier-Transform Infrared Spectroscopy (FTIR)

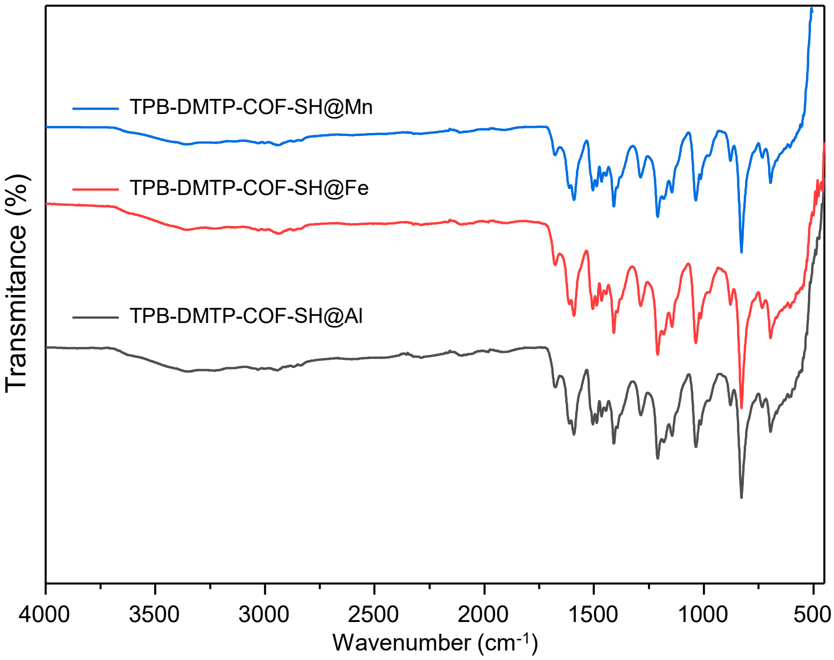

Figure S1. FTIR spectra of TPB-DMTP-COF-SH@Al, TPB-DMTP-COF-SH@Fe, and TPB-DMTP-COF-SH@Mn.

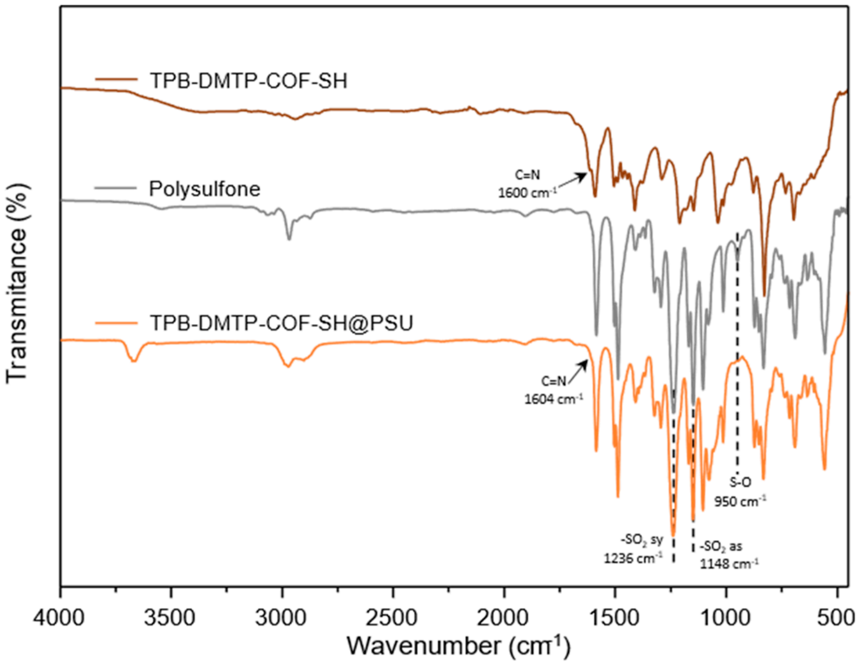

Figure S2. FTIR spectra of TPB-DMTP-COF-SH, Polysulfone and TPB-DMTP-COF-SH@PSU composite beads.

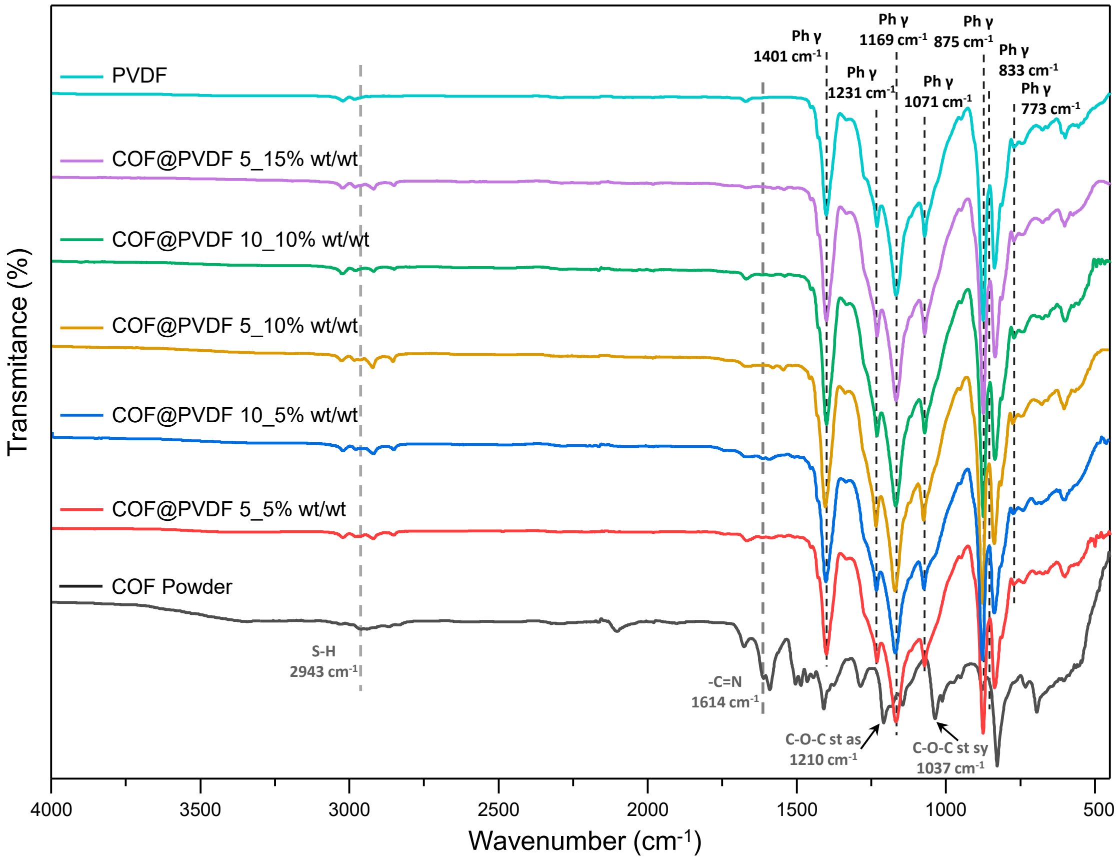

Figure S3. FTIR spectra of the pristine COF (TPB-DMTP-COF-SH), the control PVDF membrane, and COF@PVDF 5_5 %, COF@PVDF 10_5 %, COF@PVDF 5_10 %, COF@PVDF 10_10 %, COF@PVDF 5_15 %.

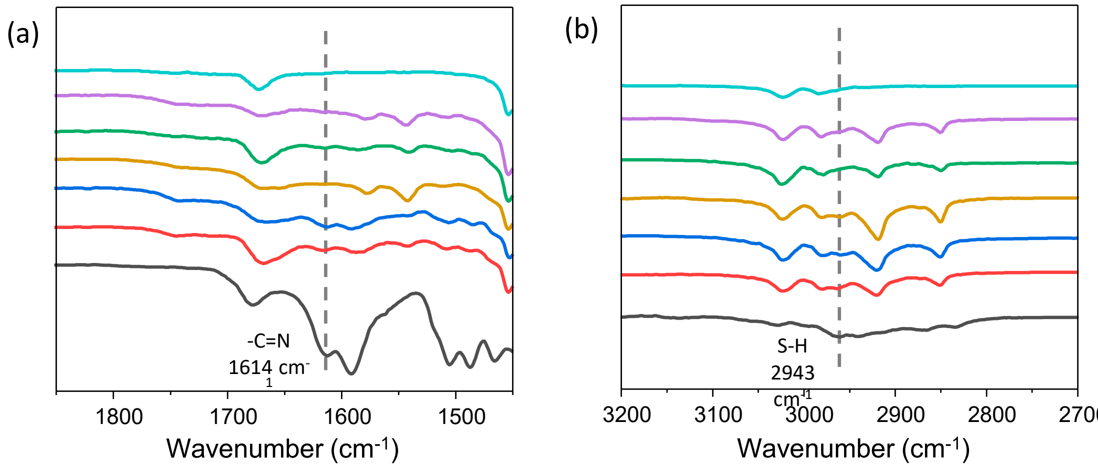

Figure S4. Comparative FTIR spectra of the prepared MMMs. (a) Close-up of the imine and (b) thiol signals of COF@PVDF 5_5 % (red), COF@PVDF 10_5 % (blue), COF@PVDF 5_10 % (yellow), COF@PVDF 10_10 % (green), COF@PVDF 5_15 % (lilac), compared to the pristine TPB-DMTP-COF-SH powder (black) and the control PVDF membrane (turquoise).

2. Elemental Analysis (EA)

Table S1. Composition of the precursors and TPB-DMTP-COF-SH obtained by elemental analysis.

Material	Molecular weight (g mol^{-1})	Formula	% C	% H	% N	% S
[C≡C]$_{0.5}$-TPB-DMTP-COF	1249	$C_{84}H_{60}N_6O_6$	79.9	4.9	6.5	0.1
TPB-DMTP-COF-N$_3$	1862	$C_{96}H_{90}N_{24}O_6S_6$	60.5	4.9	12.9	7.2
TPB-DMTP-COF-SH	1558	$C_{90}H_{75}N_{15}O_6S_3$	62.2	4.7	11.3	6.2

3. Thermogravimetric Analysis (TGA)

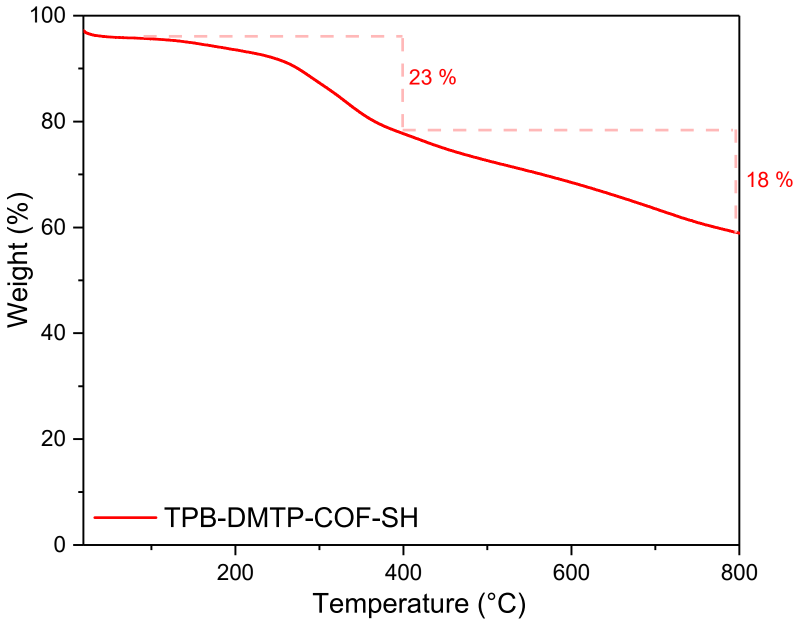

Figure S5. TGA profile of TPB-DMTP-COF-SH.

4. Additional data on the adsorption performance

Table S2. Experimental data of the adsorptive properties of TPB-DMTP-COF-SH towards different contaminants. Conditions T = 25 °C, t = 15 min.

Ion	C_i (mg L^{-1})	C_f (mg L^{-1})	m (mg)	V (mL)	Removal efficiency (%)	Q_e (mg g^{-1})
Al^{3+}	0.36	0.025	20.00	50	93	0.8375
Ca^{2+}	34.20	33.397	9.85	15	2	1.2259
Mg^{2+}	19.81	17.283	10.07	10	13	2.5058
Mn^{2+}	0.30	0.097	10.18	10	68	0.1994
Fe^{2+}	0.22	6E-04	10.28	15	99	0.3155
As^{3+}	1.02	0.990	10.00	10	3	0.0328

Table S3. Data from the pH dependence study in the removal of Al^{3+} ions of TPB-DMTP-COF-SH. Experimental conditions T = 25 °C, time = 1 h, V = 10 mL, salt: $AlCl_3$.

m (mg)	pH	C_i (mg L^{-1})	C_f (mg L^{-1})	Removal efficiency (%)	Q_e (mg g^{-1})
10.2	3.04	0.841	0.536	36	0.299
10.1	5.40	0.03	0.023	33	0.011
10.0	7.01	0.03	0.026	21	0.007
10.0	9.7	0.31	0.275	10	0.030

Table S4. Data from the pH dependence study in the removal of Fe^{2+} ions of TPB-DMTP-COF-SH. Experimental conditions T = 25 °C, time = 1 h, V = 15 mL, salt: $FeCl_2 \cdot 4H_2O$.

m (mg)	pH	C_i (mg L^{-1})	C_f (mg L^{-1})	Removal efficiency (%)	Q_e (mg g^{-1})
10.2	3.50	1.01	0.19	81	1.206
10.2	5.50	1.00	0.01	99	1.462
10.1	8.32	0.89	0.00	100	1.317
10.3	10.11	0.97	0.14	86	1.209
10.1	11.50	1.03	0.83	19	0.296

Table S5. Data from the pH dependence study in the removal of Mn^{2+} ions of TPB-DMTP-COF-SH. Experimental conditions T = 25 °C, time = 1 h, V = 12 mL, salt: $MnCl_2$.

m (mg)	pH	C_i (mg L^{-1})	C_f (mg L^{-1})	Removal efficiency (%)	Q_e (mg g^{-1})
10.0	3.44	0.131	0.131	0.38	0.001
10.1	5.96	0.117	0.117	0.43	0.001
10.1	7.50	0.145	0.122	16	0.027
10.0	10.1	0.136	0.096	29	0.048

Table S6. Data from the adsorption isotherm of TPB-DMTP-COF-SH towards Al^{3+}. Experimental conditions T = 25 °C, time = 1 h, V = 50 mL, salt: $AlCl_3$.

m (mg)	C_i (mg L^{-1})	C_f (mg L^{-1})	Removal efficiency (%)	Q_e (mg g^{-1})
0	0	0	0	0
20.10	0.095	0.01	89	0.211
20.71	0.270	0.02	92	0.604
20.00	0.360	0.025	93	0.838
20.23	0.930	0.07	92	2.126
19.70	1.500	0.44	71	2.690

Table S7. Data from the adsorption isotherm of TPB-DMTP-COF-SH towards Fe^{2+}. Experimental conditions T = 25 °C, time = 1 h, V = 25 mL, salt: $FeCl_2 \cdot 4H_2O$.

m (mg)	C_i (mg L^{-1})	C_f (mg L^{-1})	Removal efficiency (%)	Q_e (mg g^{-1})
0	0	0	0	0
10.24	0.33	0.33	99.70	0.803
10.25	0.84	0.84	99.86	2.046
10.14	0.89	0.89	99.44	1.309
10.46	1.55	1.55	99.35	3.681
10.40	2.09	2.09	98.56	4.952
10.25	2.79	2.79	96.42	6.561
10.05	3.49	3.49	92.84	8.060
10.33	3.96	3.96	89.65	8.591

Table S8. Data from the adsorption isotherm of TPB-DMTP-COF-SH towards Mn^{2+}. Experimental conditions T = 25 °C, time = 1 h, V = 12 mL, salt: $MnCl_2$.

m (mg)	C_i (mg L^{-1})	C_f (mg L^{-1})	Removal efficiency (%)	Q_e (mg g^{-1})
0	0	0	0	0
10.1	0.041	0.019	54	0.026
10.1	0.222	0.115	48	0.127
10.2	0.300	0.163	46	0.161
10.1	0.800	0.500	38	0.356
10.1	1.300	0.900	31	0.475
10.2	1.700	1.300	24	0.471

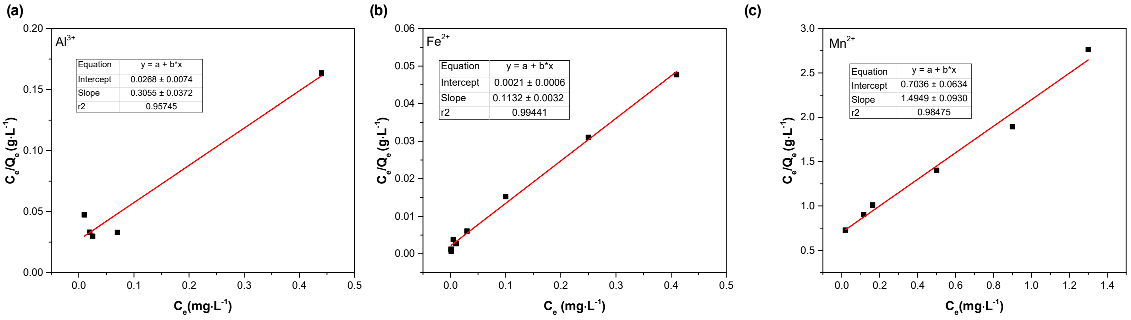

Figure S6. Adsorption isotherms of TPB-DMTP-COF-SH towards (a) Al^{3+}, (b) Fe^{2+}, and (c) Mn^{2+} fitting of Langmuir linear equation.

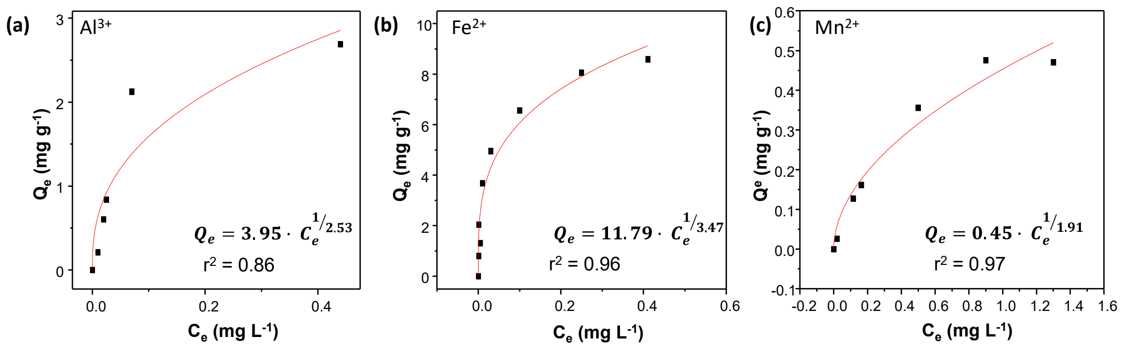

Figure S7. Adsorption isotherms of TPB-DMTP-COF-SH towards (a) Al^{3+}, (b) Fe^{2+}, and (c) Mn^{2+} fitting of Freundlich non-linear equation.

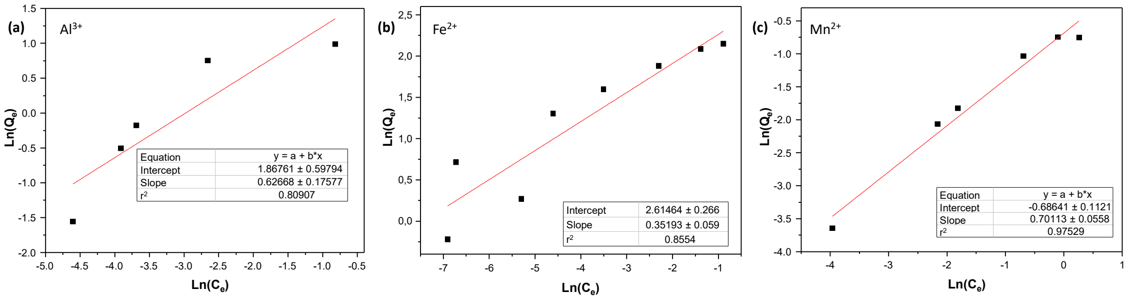

Figure S8. Adsorption isotherms of TPB-DMTP-COF-SH towards (a) Al^{3+}, (b) Fe^{2+}, and (c) Mn^{2+} fitting of Freundlich linear equation.

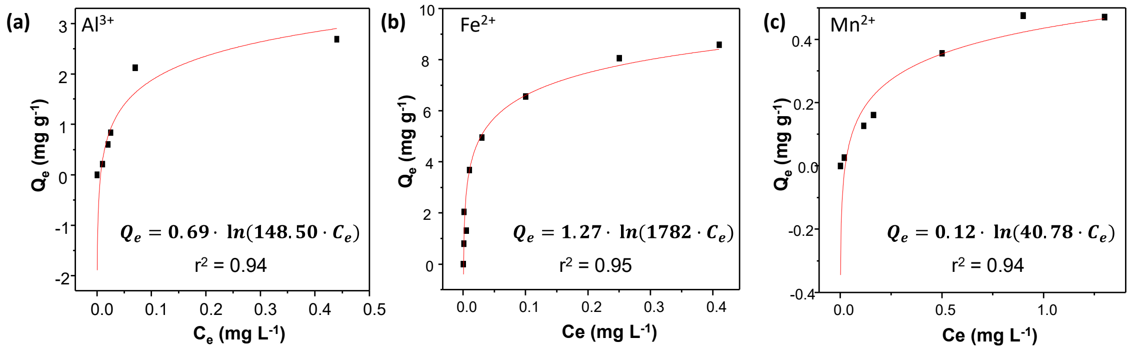

Figure S9. Adsorption isotherms of TPB-DMTP-COF-SH towards (a) Al^{3+}, (b) Fe^{2+}, and (c) Mn^{2+} fitting of Temkin equation.

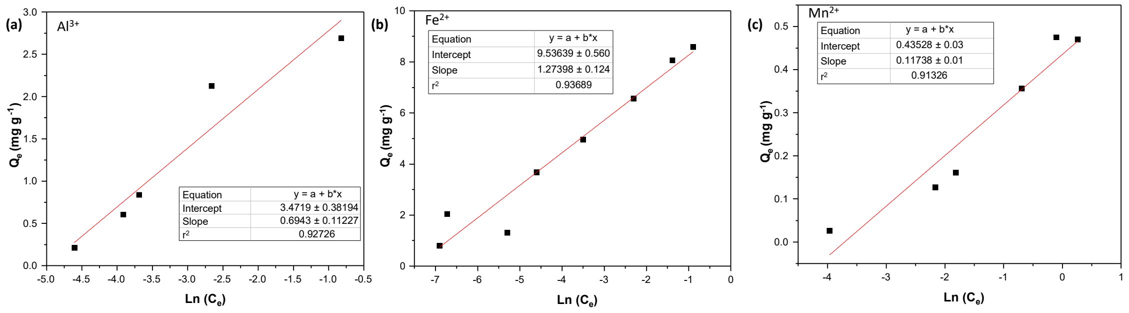

Figure S10. Adsorption isotherms of TPB-DMTP-COF-SH towards (a) Al^{3+}, (b) Fe^{2+}, and (c) Mn^{2+} fitting of Temkin linear equation.

Table S9. Experimental parameters from the adsorption isotherms of TPB-DMTP-COF-SH towards Al^{3+}, Fe^{2+} and Mn^{2+}.

Model	Parameter	Al^{3+}	Fe^{2+}	Mn^{2+}
**Langmuir**	$r^2_{, non-linear}$	0.96	0.99	0.98
	$r^2_{, linear}$	0.96	0.95	0.99
**Freundlich**	$r^2_{, non-linear}$	11.79	0.85	0.96
	$r^2_{, linear}$	0.45	0.97	0.97
**Temkin**	$r^2_{, non-linear}$	1782	0.94	0.95
	$r^2_{, linear}$	40.78	0.91	0.94

Table S10. Experimental results from the adsorption kinetic of TPB-DMTP-COF-SH towards Al^{3+}. Conditions T = 25 °C, c = 1.5 ppm, V = 10 mL, salt: $AlCl_3$.

m (mg)	t (min)	C_i (mg L^{-1})	C_f (mg L^{-1})	Removal efficiency (%)	Q_t (mg g^{-1})
**10.0**	1	1.571	1.052	33	0.519
**10.2**	3	1.571	1.037	34	0.524
**10.2**	5	1.571	1.048	33	0.513
**10.0**	10	1.571	1.034	34	0.537
**10.0**	15	1.571	0.979	38	0.592
**10.2**	30	1.571	0.970	38	0.589

Table S11. Experimental results from the adsorption kinetic of TPB-DMTP-COF-SH towards Fe^{2+}. Conditions T = 25 °C, c = 1.0 ppm, V = 15 mL, salt: $FeCl_2 \cdot 4H_2O$.

m (mg)	t (min)	C_i (mg L^{-1})	C_f (mg L^{-1})	Removal efficiency (%)	Q_t (mg g^{-1})
10.0	2	0.89	0.455	49	0.6525
9.8	5	0.89	0.500	44	0.7959
10.1	10	0.89	0.285	68	0.8985
10.2	15	0.89	0.080	91	1.1912
10.4	30	0.89	0.060	93	1.1971
10.1	60	0.89	0.005	99	1.3144
10.3	240	0.89	0.000	100	1.2961

Table S12. Experimental results from the adsorption kinetic of TPB-DMTP-COF-SH towards Mn^{2+}. Conditions T = 25 °C, c = 0.5 ppm, V = 12 mL, salt: $MnCl_2$.

m (mg)	t (min)	C_i (mg L^{-1})	C_f (mg L^{-1})	Removal efficiency (%)	Q_t (mg g^{-1})
10.0	1	0.430	0.3700	14	0.07200
10.0	3	0.430	0.3700	14	0.07200
10.1	5	0.430	0.3400	21	0.10693
10.1	10	0.430	0.3270	24	0.12238
10.0	30	0.430	0.3350	22	0.11400
10.0	60	0.430	0.3250	24	0.12600
10.0	120	0.430	0.3200	26	0.13200

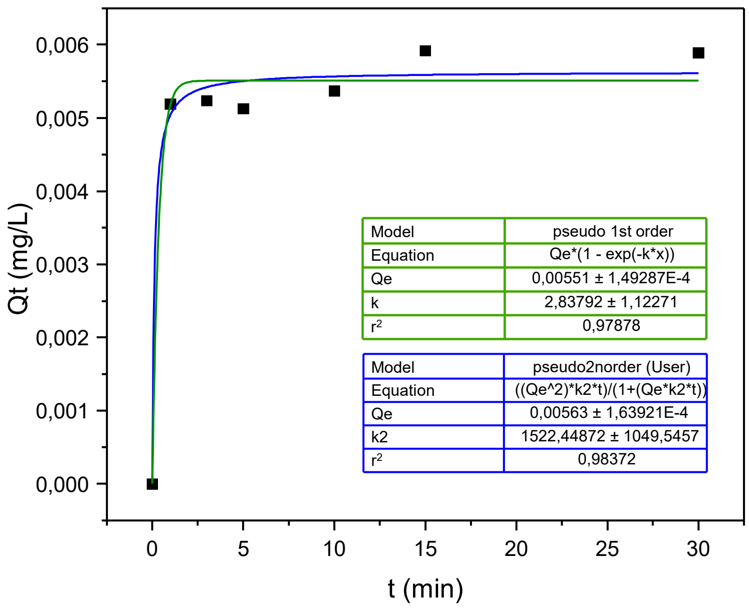

Figure S11. Adsorption kinetics of TPB-DMTP-COF-SH towards Al^{3+} fitting a pseudo-first-order and pseudo-second-order model.

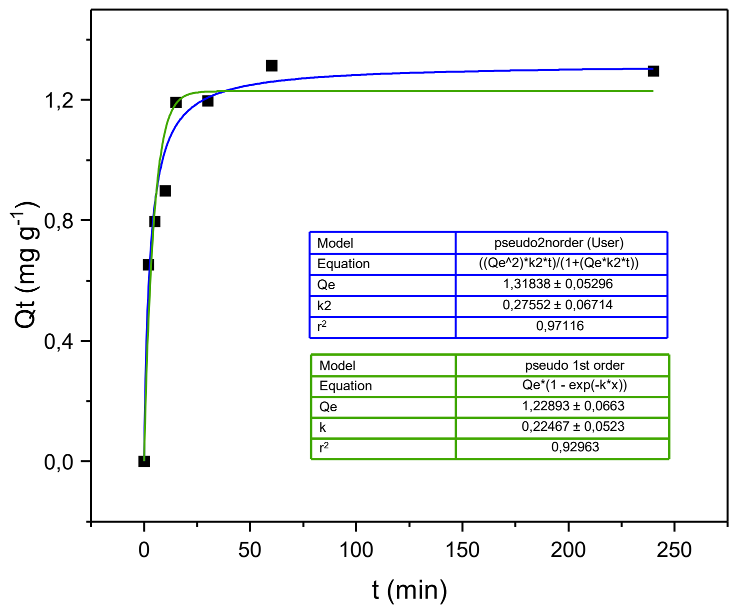

Figure S12. Adsorption kinetics of TPB-DMTP-COF-SH towards Fe^{2+} fitting a pseudo-first-order and pseudo-second-order model.

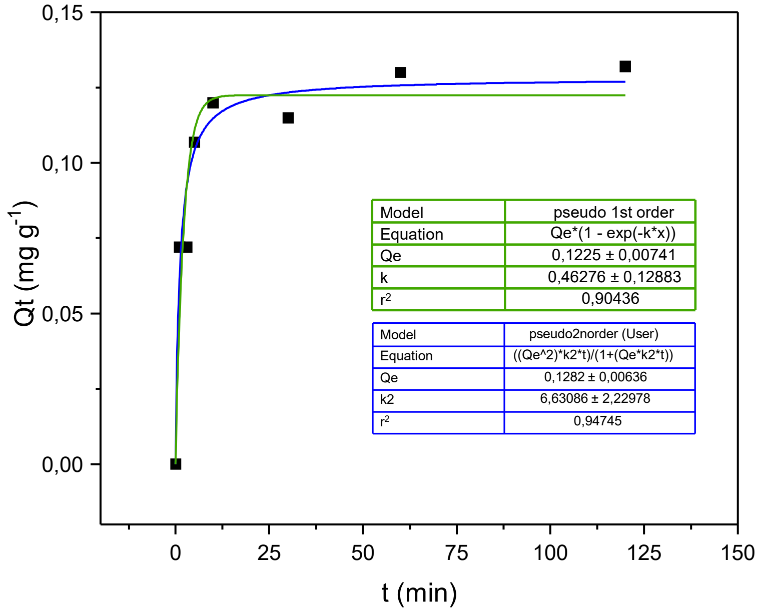

Figure S13. Adsorption kinetics of TPB-DMTP-COF-SH towards Mn^{2+} fitting a pseudo-first-order and pseudo-second-order model.

5. Preparation and performance of composite beads

Table S13. TPB-DMTP-COF-SH@PSU beads adsorption performance towards the selected ions. V_{Al} = 50 mL, V_{Fe} = 12 mL, V_{Mn} = 12 mL, t = 1 h, T = 25 °C.

Ion	m beads (mg)	m COF (mg)	C_i (mg L^{-1})	C_f (mg L^{-1})	Removal efficiency (%)	Q_e (mg g^{-1})
Al^{3+}	68.7	13.70	0.300	0.160	47	0.509
Fe^{2+}	68.5	13.74	0.650	0.450	31	0.175
Mn^{2+}	61.1	12.22	0.260	0.198	24	0.061

6. Preparation and performance of TPB-DMTP-COF-SH@PPVDF MMMs

Table S14. PVDF and COF quantities for the preparation of the different MMMs.

Membrane COF_PVDF%	mass stock solution (g)	PVDF (mg)	COF (mg)	Ø petri dish (cm)
5_5%	4	200	10.00	3.0
10_5%	4	200	20.00	3.5
5_10%	4	400	20.00	3.5
5_15%	1	150	7.50	2.5
10_10%	7.86	786	78.65	5.6

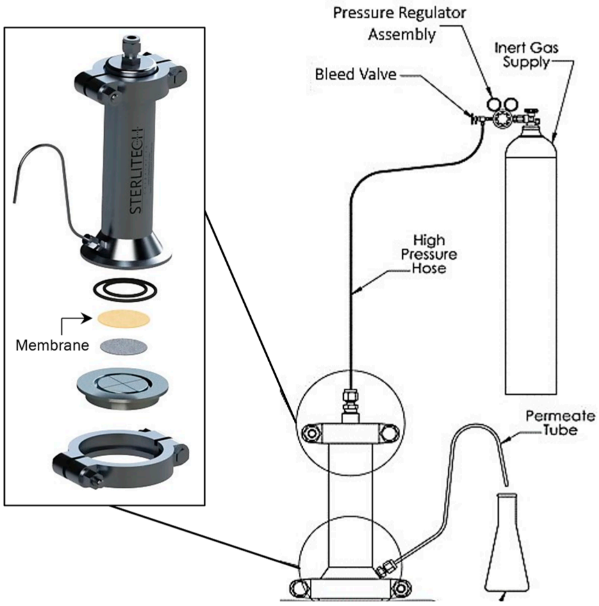

Scheme S1. Scheme of the Sterlitech HP4750 High-Pressure Stirred Cell.

This ultrafiltration module has a capacity of 300 mL and can be used at a maximum pressure of 69 bar. The membrane is placed at the bottom, secured with the

cell bottom lid and the clamps, and the solution is forced to pass tangentially through the membrane with the help of an attached N_2 gas tank. The permeate is collected for 1 minute under constant pressure, and the J is calculated from Eq. 1, considering the active membrane area used for the experiment.

$$J = \frac{V}{A \cdot t} \qquad \text{Eq.1}$$

where V (L) is the volume, A (m^2) is the area and t (h) is the time.

Table S15. Volumetric fluxes of the prepared membranes and characteristics. Experimental conditions: t = 0.02 h, area = 0.000137 m^2.

Sample	J (L m^{-2} h^{-1})	V (mL)	P (Bar)	% PVDF	% COF	Thickness (mm)
PVDF	438	1.0	2	5	0	0.15
PVDF	350	0.8	2	10	0	0.18
PVDF	175	0.8	2	15	0	0.30
5_5 %	1926	4.4	2	5	5	0.13
10_5 %	3289	12.2	2	5	10	0.24
5_10 %	613	1.4	2	10	5	0.17
10_10 %	525	1.2	1	10	10	0.20

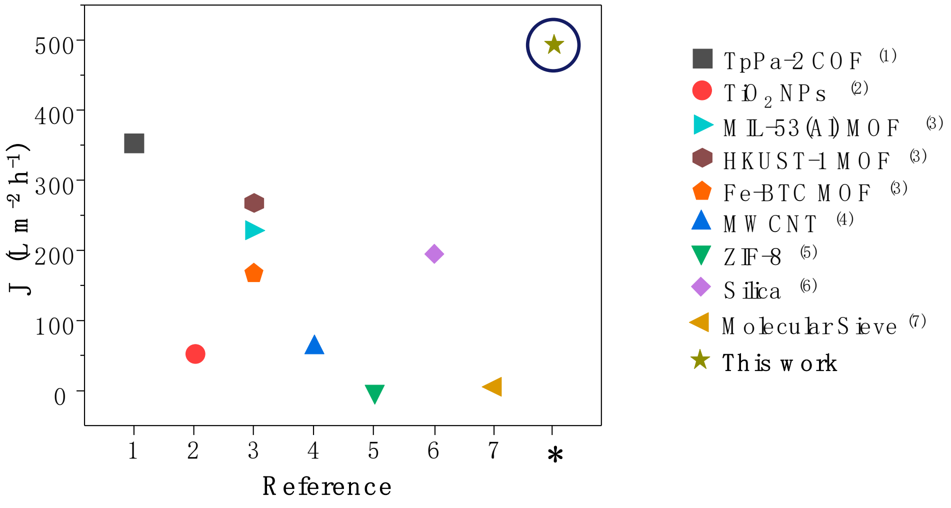

Figure S14. Comparison of operating flows with other porous materials.

7. References

1. Xu, L.; Xu, J.; Shan, B.; Wang, X.; Gao, C. TpPa-2-Incorporated Mixed Matrix Membranes for Efficient Water Purification. *J Memb Sci* **2017**, *526*, 355–366.
2. Teow, Y.H.; Ooi, B.S.; Ahmad, A.L. Fouling Behaviours of PVDF-TiO2 Mixed-Matrix Membrane Applied to Humic Acid Treatment. *Journal of Water Process Engineering* **2017**, *15*, 89–98.
3. Lee, J.-Y.; Tang, C.Y.; Huo, F. Fabrication of Porous Matrix Membrane (PMM) Using Metal-Organic Framework as Green Template for Water Treatment. *Sci Rep* **2014**, *4*, 3740.
4. Majeed, S.; Fierro, D.; Buhr, K.; Wind, J.; Du, B.; Boschetti-de-Fierro, A.; Abetz, V. Multi-Walled Carbon Nanotubes (MWCNTs) Mixed Polyacrylonitrile (PAN) Ultrafiltration Membranes. *J Memb Sci* **2012**, *403–404*, 101–109.
5. Duan, J.; Pan, Y.; Pacheco, F.; Litwiller, E.; Lai, Z.; Pinnau, I. High-Performance Polyamide Thin-Film-Nanocomposite Reverse Osmosis Membranes Containing Hydrophobic Zeolitic Imidazolate Framework-8. *J Memb Sci* **2015**, *476*, 303–310.
6. Kumar, S.; Guria, C.; Mandal, A. Synthesis, Characterization and Performance Studies of Polysulfone/Bentonite Nanoparticles Mixed-Matrix Ultra-Filtration Membranes Using Oil Field Produced Water. *Sep Purif Technol* **2015**, *150*, 145–158.
7. Qadir, D.; Mukhtar, H.; Keong, L.K. Synthesis and Characterization of Polyethersulfone/Carbon Molecular Sieve Based Mixed Matrix Membranes for Water Treatment Applications. *Procedia Eng* **2016**, *148*, 588–593.
